# A New Splice Variant of the Major Subunit of Human Asialoglycoprotein Receptor Encodes a Secreted Form in Hepatocytes

**DOI:** 10.1371/journal.pone.0012934

**Published:** 2010-09-23

**Authors:** Jia Liu, Bin Hu, Yan Yang, Zhiyong Ma, Yuan Yu, Shenpei Liu, Baoju Wang, Xiping Zhao, Mengji Lu, Dongliang Yang

**Affiliations:** 1 Division of Clinical Immunology, Tongji Medical College, Tongji Hospital, Huazhong University of Science and Technology, Wuhan, Hubei Province, China; 2 Experimental Medicine Center, Tongji Medical College, Tongji Hospital, Huazhong University of Science and Technology, Wuhan, Hubei Province, China; 3 Department of Microbiology, Tongji Medical College, Huazhong University of Science and Technology, Wuhan, Hubei Province, China; 4 Institute of Virology, Medical School, Duisburg-Essen University, Essen, Germany; The University of Hong Kong, Hong Kong

## Abstract

**Background:**

The human asialoglycoprotein receptor (ASGPR) is composed of two polypeptides, designated H1 and H2. While variants of H2 have been known for decades, the existence of H1 variants has never been reported.

**Principal Findings:**

We identified two splice variants of ASGPR H1 transcripts, designated H1a and H1b, in human liver tissues and hepatoma cells. Molecular cloning of ASGPR H1 variants revealed that they differ by a 117 nucleotide segment corresponding to exon 2 in the ASGPR genomic sequence. Thus, ASGPR variant H1b transcript encodes a protein lacking the transmembrane domain. Using an H1b-specific antibody, H1b protein and a functional soluble ASGPR (sASGPR) composed of H1b and H2 in human sera and in hepatoma cell culture supernatant were identified. The expression of ASGPR H1a and H1b in Hela cells demonstrated the different cellular loctions of H1a and H1b proteins at cellular membranes and in intracellular compartments, respectively. In vitro binding assays using flourescence-labeled sASGPR or the substract ASOR revealed that the presence of sASGPR reduced the binding of ASOR to cells. However, ASOR itself was able to enhance the binding of sASGPR to cells expressing membrane-bound ASGPR. Further, H1b expression is reduced in liver tissues from patients with viral hepatitis.

**Conclusions:**

We conclude that two naturally occurring ASGPR H1 splice variants are produced in human hepatocytes. A hetero-oligomeric complex sASGPR consists of the secreted form of H1 and H2 and may bind to free substrates in circulation and carry them to liver tissue for uptake by ASGPR-expressing hepatocytes.

## Introduction

The asialoglycoprotein receptor (ASGPR), a well-characterized hepatic lectin, is responsible for the selective binding and internalization of galactosel/N-acetylgalactosamine-terminating glycoproteins by hepatic parenchymal cells [1], [2]. The activity of this receptor remains a key factor in the development and administration of glycoprotein pharmaceuticals, yet its biological function has remained elusive. Recent studies suggest a function of ASGPR in hemostatic modulation in response to a reduced sialylation state of platelets, vWF and possibly other blood components [3]. Human ASGPR is a hetero-oligomer composed of a major subunit (H1) and a minor subunit (H2) which are encoded by two different genes expressed in a molar ratio of ∼3∶1 [4]–[6]. Both subunits are type II single-spanning membrane proteins. Amino acid (aa) sequence indicates that the ASGPR H1 subunit contains a 40 aa N-terminal cytoplasmic domain, a ∼20 aa single-pass transmembrane domain (TMD), an ∼80 aa extracellular stalk (oligomerization) region, and a ∼140 aa functional calcium-dependent acid carbohydrate recognition domain (CRD) [7].

Three naturally occurring ASGPR H2 splice variants have been identified, designated H2a, H2b, and H2c [4], [8]. Compared to the smallest splice variant, H2c, the largest isoform H2a contains a 57 nucleotide (nt) insert encoding part of the cytoplasmic domain and a 15 nt insert encoding a 5 aa sequence near the junction between the TMD and the ectodomain [4]. The H2a isoform is not incorporated into native ASGPR complexes on the cell surface. Rather, the 5 aa sequence encoded by the 15 nt insert, serves as a cleavage signal that results in proteolysis and the secretion of the entire H2a ectodomain [9]. Thus, the soluble CRD of the ASGPR is present in human serum [10]. ASGPR H2b or H2c isoforms lacking the 5 aa sequence insert are not proteolytically cleaved and oligomerize with H1 subunits to form native human ASGPR on hepatocytes.

While variants of H2 have been known for decades, the existence of H1 variants has never been reported. During a recent attempt to clone cDNAs of the human ASGPR H1 subunits, we made an unanticipated discovery of an H1 splice variant in human liver tissues and in the human hepatoma cell lines HepG2 and Huh7. We named the original sequence of ASGPR H1 and its novel variants H1a and H1b, respectively, in accordance with the accepted terminology of H2 variants [4], [8].

## Materials and Methods

### Reagents

Unless otherwise noted, all chemicals were purchased from Sigma-Aldrich (St. Louis, MO). Restriction enzymes, T4 DNA ligase, and Taq DNA polymerase were purchased from TaKaRa Biotechnology Co. Ltd. (Dalian, China). TRIzol Reagent, SuperScript II reverse transcriptase, and all cell culture products were purchased from Invitrogen (Carlsbad, CA). Monoclonal mouse anti-hemagglutinin (HA), goat anti-mouse HRP-IgG, and goat anti-rabbit HRP-IgG antibodies were purchased from DAKO Corp. (Glostrup, Denmark). Plasmids pXF3H and pXF1E, mammalian expression vector with the cytomegalovirus immediate-early promoter and hemagglutinin (HA) tag or enhanced green fluorescent protein (EGFP) tag individually, were described previously [11].

### Human liver tissues and sera

Human liver tissues from patients were obtained from the Department of Liver Surgery Center of Tongji Hospital and stored at −80°C. Human sera for sASGPR purification were collected from volunteers by the Division of Clinical Immunology, Tongji Hospital and stored at −80°C. This study had institutional review board approval. All patients and volunteers who enrolled in this study provided informed written consent according to the Helsinki declaration.

**Cell culture**

HeLa [11], Huh7 [12], HepG2 [13], and HepG2.2.15 cells [13] with hepatitis B virus (HBV) replication were grown in DMEM/high glucose supplemented with 10% fetal bovine serum and were grown at 37°C in H2O-saturated 95% air, 5% CO2. Cells were seeded into 35 mm plastic dishes for 24 hours before proceeding with experiments. Huh7.5.1 cells were kindly provided by Professor Xinwen Chen (Wuhan Institute of Virology, Chinese Academy of Sciences). For infection with hepatitis C virus, the cultured JFH-1 strain was used. A complete infection of Huh7.5.1 cells was reached after 6 days post infection [12].

### Isolation of total RNA

Total cellular RNA was extracted from human liver and cultured cells with TRIzol reagent (used according to the manufacturer's instructions). Briefly, 100 mg of human liver or 5×106 cells were lysed in 1 ml of TRIzol reagent at 22°C for 5 min. Chloroform (200 ml) was added to the lysates, and samples were mixed by vortexing. Samples were centrifuged at 12,000 *g* for 15 min at 4°C. The resulting aqueous phase was transferred to an Eppendorf tube containing 0.5 ml of isopropyl alcohol. After a 10 min incubation, the precipitated RNA was pelleted by centrifugation at 12,000 *g* for 10 min at 4°C. The RNA pellet was resuspended, washed in 1 ml of 75% ethanol, and then pelleted. The pellet was air-dried and dissolved in 50 µl of water. Purified RNA was routinely checked on formaldehyde agarose gels.

### Reverse transcription PCR

cDNA templates were synthesized from 1 µg of total RNA, using SuperScript II reverse transcriptase and an oligo(dT) primer according to the manufacturer's instructions. PCR was performed in a single 50 µl reaction. The PCR program consisted of incubation for 10 min at 94°C, followed by 35 cycles of 94°C for 60 sec, 55°C for 60 sec, and 72°C for 80 sec, and a final cycle of 10 min at 72°C. PCR products were visualized on a 1.0% agarose gel stained with ethidium bromide. The primers used for cloning ASGPR H1 variant cDNAs were: 5′-CCG GAA TTC ATG ACC AAG GAG TAT CAA GAC-3′ (P1, sense, nt 180–200), 5′-ATT TGC GGC CGC CTT CCC TTA AAA TCC TAG ATG-3′ (P2, antisense, nt 1145–1165), and the primers used to detect H1 variants were: 5′-GAG TAT CAA GAC CTT CAG CAT C-3′ (P3, sense, nt 189–210), 5′-CTT CAT CTT TCT TCC CAC ATT-3′ (P4, antisense, nt 468–488). H2c cDNA was amplified by nest PCR. The outer primers used for cloning ASGPR H2c cDNAs were: 5′-CAG CCC TCA GAG CAA CCT-3′ (sense, nt 238–255) and 5′-CCA TTG AAG AGG CTG ACG A-3′ (antisense, nt 1443–1461), the inner primers were: 5′-CCG GAA TTC ATG GCC AAG GAC TTT CAA G-3′ (sense, nt 305–323) and 5′-ATT TGC GGC CGC CAA AAT CCT CAA CAG AGA AGC-3′ (sense, nt 1289–1309). 20 cycles of first PCR and 35 cycles of second PCR were performed. The nucleotide positions are numbered according to the mRNA sequence of Genbank accession number NM_001671 and NM_080912. The tagged restriction enzyme cleavage sites are underlined.

### Plasmid construction and sequencing

PCR products corresponding to ASGPR H1a, H1b and H2c cDNAs were excised from the agarose gel, purified using a Gel Extraction Kit (OMEGA, Victoria BC, Canada), and H1a and H1b were subcloned into pcDNA3.1(+)/neo via the *EcoR*I and *Not*I restriction sites to yield plasmids pcDNA3.1-H1a and pcDNA3.1-H1b. H2c cDNA was subcloned into pcDNA3.1 (+)/zeo (Invitrogen) via the *Eco*RI and *Not* I sites. The nucleotide sequences of the ASGPR H1-inserts were verified by DNA sequencing (Sangon Biotechnology Co. Ltd., Shanghai, China). The ASGPR H1a and H1b cDNAs were then subcloned into pXF3H or pXF1E via the *EcoR*I and *Xba*I sites to yield plasmids pXF3H-H1a, pXF1E-H1a, pXF3H-H1b and pXF1E-H1b respectively, to generate recombinant proteins of H1a and H1b with HA tag or EGFP tag, for celluar location analysis and WB analysis. H1a was also subcloned into pIRES/neo (CLONTECH) via the *Sal*I *and EcoR*I sites to generate plasmid pIRES-H1a for stable ASGPR expressing cell line construction.

### Real-time fluorescence quantitative PCR (FQ-PCR)

The primer sequences used in real-time FQ-PCR were as follows: sense primer for ASGPR H1a: 5′-TGC TGC TTG TGG TTG TCT G-3′ (P5, nt 331–349), sense primer for ASGPR H1b: 5′-GCT CAG AAA AGA CTC CCA G-3′ (P6, nt 239–249 combined with nt 367–374), antisense primer for both ASGPR H1a and H1b: 5′-CTT CAT CTT TCT TCC CAC ATT-3′ (P4, nt 468–488). Nucleotide positions were numbered according to the mRNA sequence of Genbank accession number NM_001671. Standard curves for ASGPR H1a and H1b cDNA quantification were made with serial dilutions (103–109 copies) of plasmids pcDNA3.1-H1a and pcDNA3.1-H1b, respectively. For quantitative real-time PCR analysis of ASGPR H1a and H1b mRNA expression levels, the LightCycler system (Roche, Rotkreuz, Switzerland) and a Realtime PCR Master Mix Kit (TOYOBO Co. Ltd., Osaka, Japan) were used according to the manufacturer's instructions.

### Preparation of anti-ASGPR H1b antibodies

ASGPR H1b-specific epitopes were predicted online (http://www.imtech.res.in/raghava/bcipep). The peptide SDHHQLRKDSQLQE, corresponding to amino acid residues 16–29 of ASGPR H1b, and peptide-KLH conjugate were synthesized individually and then used in antibody preparation. Peptide synthesis and antibody preparation were accomplished by Proteintech Group, Inc., Wuhan, China. Antibody titer and specificity were determined by indirect ELISA using a single peptide antigen. The specificity of one mouse antiserum to ASGPR H1b was also confirmed by Western blot.

### Transfection

The expression plasmids pXF3H-H1a, pXF1E-H1a, pXF3H-H1b, and pXF1E-H1b were transfected individually or cotransfected into HeLa cells with Lipofectamine 2000 (Invitrogen, Carlsbad, CA) according to the manufacturer's instructions. Transfected cells were collected 36 h later and lyzed in sample buffer for SDS PAGE and Western blot analysis.

### Immunofluorescence staining

In brief, pXF3H-H1a and pXF1E-H1b cotransfected cells were fixed in 50% methanol for 15 min at 4°C and dried at room temperature, then incubated with mouse anti-HA antibody for 1 h at 37°C. PBS wash for 3 times, then cells were incubated with PE labeled goat anti-mouse antibody for 1 h at 37°C. PBS wash for 3 times.

### Immunohistochemistry (IHC)

*IHC* was performed on 4 µm sections cut from paraffin-embedded human liver tissue specimens. Sections were deparaffinized in xylene, dehydrated in ethanol, washed in phosphate-buffered saline (PBS), and treated with 0.3% H2O2 in methanol for 30 min to block endogenous peroxidase activity. Sections were blocked with 10% normal goat serum in PBS for 10 min at room temperature and incubated overnight at 4°C with primary antibodies. After washing in PBS, the sections were incubated with Envision solution (a cocktail of peroxidase-labeled polymer conjugated to goat anti-rabbit and goat anti-mouse immunoglobulins in Tris–HCl buffer; DAKO Corp., Glostrup, Denmark) for 45 min at room temperature. The color reaction was incubated in 3, 3-diaminobenzidine. Sections were counterstained with hematoxylin.

### Purification of soluble ASGPR (sASGPR)

sASGPR in normal human sera or HepG2 cell supernatants were purified by lactose-agarose affinity chromatography, modified from a protocol for purification of ASGPR from liver tissues [14]. Briefly, a column containing a ∼10 ml bed volume of lactose-agarose (Sigma, St. Louis, MO) was equilibrated with wash buffer I (50 mM Tris-Cl, pH 7.8, 0.5% Lubrol, 50 mM CaCl2, 1.25 M NaCl) at 4°C. 50 ml normal human pooled sera or 50 ml HepG2 cell supernatant was mixed with 50 ml lysis buffer (100 mM Tris-Cl, pH 7.8, 2% Lubrol, 2.5 M NaCl), stirred for 1 h at 4°C, and then centrifuged at 25,000 *g* for 30 min at 4°C. Supernatant was removed and incubated with 5 ml 1 M CaCl2 for 30 min on ice. Following a second centrifugation, supernatant was applied to a 10 ml lactose-agarose column at a flow rate of 25–50 ml per hour at 4°C. The column was washed with 50 ml wash buffer I and eluted with 42.5 ml elution buffer (20 mM sodium acetate, pH 5.4, 0.1% Lubrol, 1.25 M NaCl). The eluate was readjusted with 5 ml of 1 M Tris-Cl, pH 7.8 and 2.5 ml 1 M CaCl2 and applied to a 1 ml lactose-agarose column at a flow rate of 25–50 ml per hour at 4°C. After washing with 5 ml wash buffer II (50 mM Tris-Cl, pH 7.8, 0.1% Lubrol, 50 mM CaCl2, 1.25 M NaCl), the column was eluted with 10 ml elution buffer. The eluted proteins were concentrated to 15 µM with Amicon Ultra-4 Centrifugal Filter Devices, 5,000 NMWL (Millipore, Billerica, MA) and stored at −80°C.

### Silver stain

Approx. 500 ng purified protein was separated by 12% SDS-polyacrylamide gel electrophoresis (PAGE) and stained using a Silver Stain Plus kit (Bio-Rad Laboratories, Hercules, CA).

### Western blot analysis

Purified sASGPR protein or total protein from transfected cells was separated on 12% SDS-PAGE gels and transferred to nitrocellulose membranes. Fat-free milk powder solution (10% w/v) was used for blocking. The membrane was then incubated with the appropriate primary antibody overnight at 4°C, washed three times with PBST, and incubated with a 1∶5,000 dilution of horseradish peroxidase-conjugated goat anti-rabbit IgG or goat anti-mouse IgG. After washing, the proteins were detected by using Lumi-light Western blotting detection kit (Roche, Rotkreuz, Switzerland) according to the manufacturer's instructions.

### Construction of stably transfected cell lines expressing recombinant human ASGPR

HeLa cells were cotransfected with plasmid constructs coding for two full length subunits of human hepatic ASGPR (H1a and H2c) that have both been previously shown to be targeted to the plasma membrane in HepG2 cells [15]. Briefly, plasmids pIRES-H1a (neo) and pcDNA3.1-H2c (zeo) were transfected to HeLa cells simultaneously using Lipofectamine 2000. At day 3 after transfection, cells were passaged and grown under G418 and zeocin selection. Upon several passages, some stable ASGPR expressing HeLa cell clones were obtained. The expressing of ASGPR at the mRNA and protein levels were confirmed by reverse transcription PCR and immunofluorescence, respectively. The function of the ASGPR of these clones was also confirmed by ASOR binding and internalization assay. One stable ASGPR expressing Hela cell line 4-1-6 was chosen for further study.

### In vitro ligand binding

The preparation of ASOR has been described previously [16]. Briefly, 10 mg of human orosomucoid was dissolved in 1 mL of 50 mM acetate buffer, pH 5, and incubated with 0.6 IU of immobilized neuraminidase for 3 hours at 37°C. Then, neuraminidase was removed by centrifugation. The removal of sialic acid was complete as measured by the thiobarbituric acid assay. The Alexa Fluor 647 labeled ASOR and sASGPR were prepared by using Alexa Fluor® 647 Protein Labeling Kit according to the manufacturer's instruction. HeLa, Huh7, and 4-1-6 cells were seeded into 24-well plate, and grown to over 90% confluency. The cells were serum-starved for 20 min before the binding assay, and then incubated with correspondingly labeled protein for 40 min at 37°C. The medium were removed and the cells were chilled on ice, then washed twice with ice-cold PBS. The cells were detached from the plate by trypsin digestion and the MFI of the cells were analyzed by FACS.

## Results

### Two different H1 transcripts are present in human liver tissues and cultured hepatoma cells

**Figure 1:**
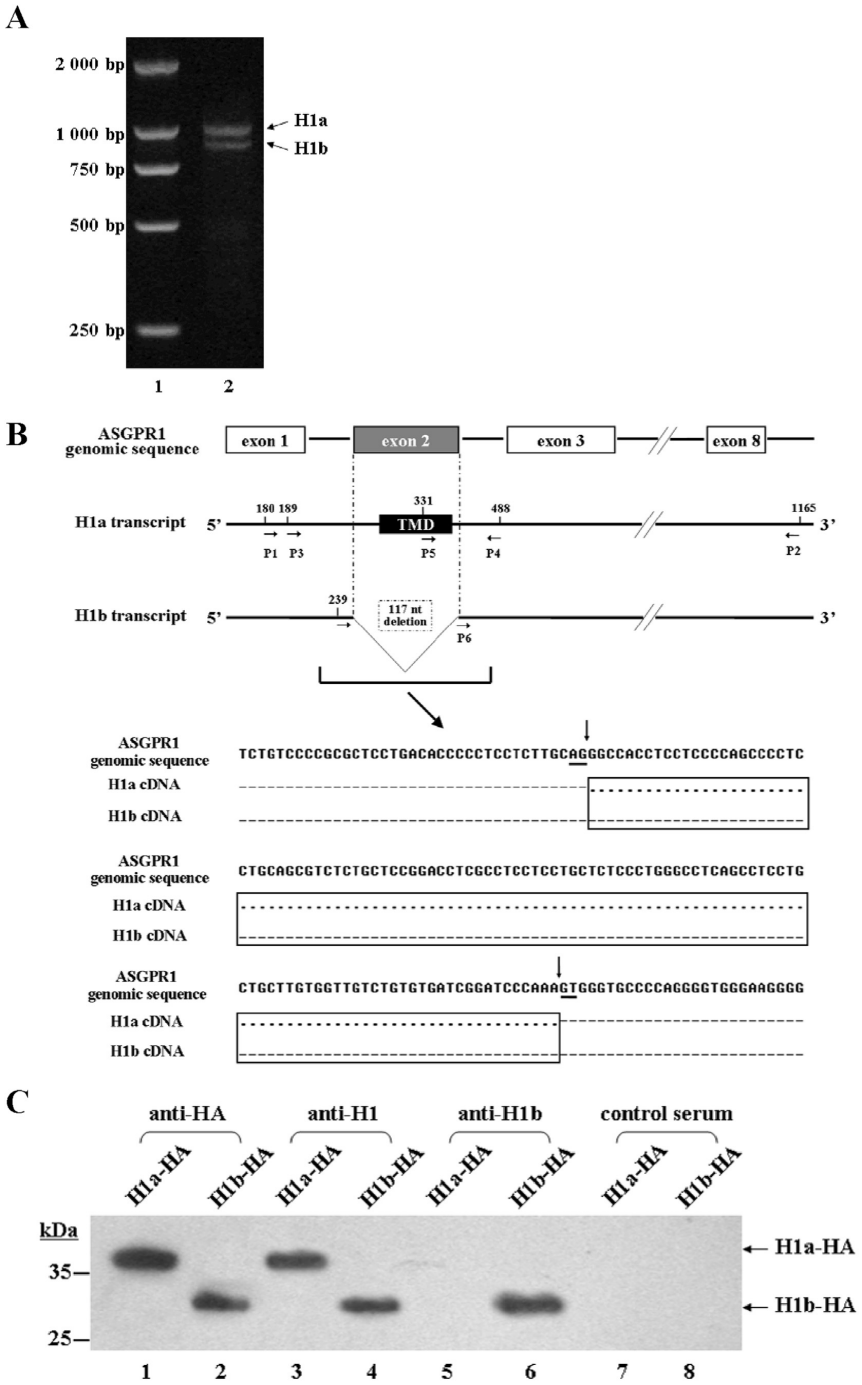
Sequence analysis of splice variants of ASGPR H1 and identification of polyclonal antibody that can recognize ASGPR H1b specifically. (A) Amplification of ASGPR H1a and H1b cDNAs from human liver tissue. Lane 1: DNA ladder. Lane 2: RT-PCR products of total RNA from human liver tissue. (B) Sequence comparison of ASGPR H1a and H1b transcripts. Upper part: the relevant parts of ASGPR H1 genomic and mRNA sequences are indicated. The structure of the genomic sequence encoding ASGPR H1 with relevant exons was shown. The 117 bp deletion in ASGPR H1b mRNA sequence corresponds exactly to the second exon (gray box) of the genomic sequence of ASGPR H1. The primers P1 to P6 used for ASGPR H1 cDNA cloning and for detection of the variant sequences are marked at corresponding position. Lower part: the sequence alignment of the genomic sequence of ASGPR H1 and cDNA sequences of H1a and H1b shows the boundaries of the alternatively spliced 117 nt region in the genomic sequence and the consensus splice donor/acceptor sites (underlined). The difference between the mRNA sequences of ASGPR H1a and H1b is boxed. Identical nucleotides are indicated by dots; missing nucleotides are indicated by dashes. (C) Specificity analysis of polyclonal antibodies by western blot. Cell lysates of H1a-HA (lane 1, 3, 5, 7) or H1b-HA (lane 2, 4, 6, 8) transfected cells were separated by SDS-PAGE. Primary antibodies are used as follows: lanes 1 and 2: anti-HA; lanes 3 and 4: anti-H1; lanes 5 and 6: anti-H1b; lanes 7 and 8: non-immunized mouse serum. The positions of H1a-HA and H1b-HA are indicated by arrows.

A number of complete ASGPR H1 cDNAs were cloned by RT-PCR amplification from total RNA of normal adult human liver tissue. A cDNA sequence of 1,007 bp was expected, based on the ASGPR H1 mRNA sequence published in Genbank (NM_001671). However, two ASGPR H1 cDNA species were obtained and could be separated on an agarose gel (Fig. 1A). These two ASGPR H1 cDNAs were cloned and sequenced. The longer sequence, designated ASGPR H1a, was identical to the known H1 sequence. The shorter sequence, now designated ASGPR H1b, differs from the H1a by the deletion of 117 nucleotides near the 5′ end. The position of the 117 nt deletion in ASGPR H1b corresponds to nt 250–366 in H1a, starting the nt numbering according to mRNA sequence NM_001671 (Fig. 1B). An alignment of the ASGPR H1a and H1b sequences with the genomic sequence of ASGPR1 (Genbank accession number NM_000017) revealed that the 117 nt deletion in H1b corresponds to the second exon of the ASGPR1 genomic sequence. The typical AG/GT 5′ consensus splice donor and acceptor sites were found at each end of Exon 2 (Fig. 1B), strongly suggesting that ASGPR H1b is generated by alternative splicing of ASGPR mRNA.

To distinguish the ASGPR H1a and H1b transcripts, primers were designed to amplify small fragments of 300 and 183 bp for H1a and H1b transcripts, respectively, allowing clear separation of the fragments with agarose gel electrophoresis. These two cDNA fragments were excised from the gel and sequenced to verify that they were derived from ASGPR H1a and H1b transcripts. Both ASGPR H1a and H1b transcripts were present in human hepatoma cell lines HepG2 and Huh7 (Figure S1A). ASGPR H1b transcripts were also found in liver tissues from patients with liver diseases. A total of 35 livers were examined, incl. 3 normal livers, 1 chronic hepatitis, 2 cirrhosis, 1 Hepatic hemangioma tissue and 28 hepatocellular carcinoma (HCC). In all samples both H1a and H1b were expressed. The non-hepatic cell line HeLa did not express ASGPR, as reported, and was used as a control [6].

A real-time RT-PCR assay was used to quantify ASGPR H1a and H1b transcripts. The real time RT-PCR amplified short fragments of 158 bp and 133 bp of ASGPR H1a and H1b, respectively (Figure S1B). The expression levels of ASGPR H1a and H1b in different liver tissues and cell lines were measured and presented as copy numbers normalized against beta-actin mRNAs (Table 1). The data indicated that both splice variants of ASGPR mRNAs were expressed in normal liver tissues and tumor tissues at variable levels.

**Table 1:** Quantitation of the expression of ASGPR H1 splice variants.

Samples			H1a copy no. (per 106 β-actin)	H1b copy no. (per 106 β-actin)
Liver tissues	Patient 1	peritumor	55,169	6,992
		tumor	14,378	3,401
	Patient 2	peritumor	97,396	156,041
		tumor	7,867	6,801
	Patient 3	peritumor	85,971	86,569
		tumor	1,400	2,050
	Patient 4	peritumor	47,366	25,208
		tumor	13,230	13,792
Normal Hepatic tissue	Patient 5		558,644	48,698
Hepatic hemangioma tissue	Patient 6		128,514	32,804
Hepatic cell lines	HepG2		43,586	937
	Huh7		11,359	1,289

### Detection of ASGPR H1b

The amino acid sequences of ASGPR H1a and H1b are predicted according to the cDNA sequence. ASGPR H1a and H1b differ in 40 aa that correspond to aa 24–63 in ASGPR H1a. This region contains the ASGPR H1a transmembrane domain (aa 20–40) and is essential for the localization of ASGPR complexes in the cell membrane.

Based on the predicted sequence of ASGPR H1a and H1b, a specific antibody directed to a peptide comprising aa 16–29 (anti-H1b) was generated (Figure S2A). The antiserum recognized the ASGPR H1b specific peptide, but not unrelated peptides, at dilutions >1∶100,000 in ELISA (Figure S2). Moreover, anti-H1b was shown to specifically recognize ASGPR H1b, but not H1a, by Western blot (Fig. 1C).

ASGPR H1a and H1b cDNAs were subcloned into pcDNA3.1(+)/neo and transfected into HeLa cells. The expression of both HA-tagged proteins with molecular weights of ∼28 kDa was detected by Western blot with anti-HA antibody and a commercial antibody to ASGPR H1 (Santa Cruz Biotechnology, Santa Cruz, CA). Anti-H1b recognized recombinant ASGPR H1b-HA but not recombinant H1a-HA (Fig. 1C).

### Expression and location of ASGPR H1b in human liver tissues and transfected cells

**Figure 2:**
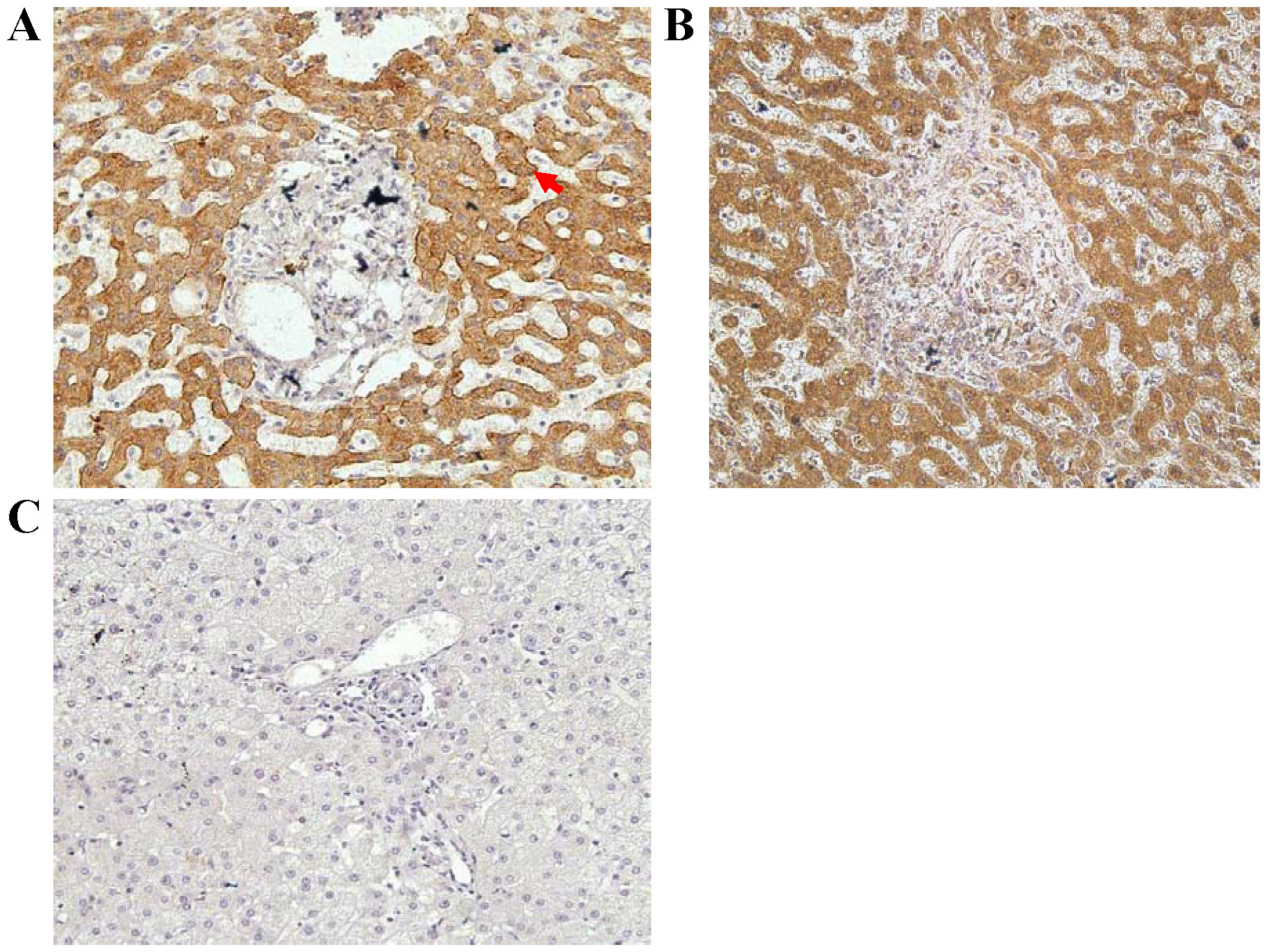
Localization of ASGPR H1b protein in human liver tissue by immunohistochemistry. (A) Paraffin sections of human liver tissue were stained with anti-H1 antibody. Staining of both the surface of the cellular membrane (red arrow) and the cytoplasm are observed. (B) Paraffin sections of human liver tissue were stained with anti-H1b antibody. Only cytoplasmic staining is observed. (C) Negative control, stained with sera from non-immunized mice.

**Figure 3:**
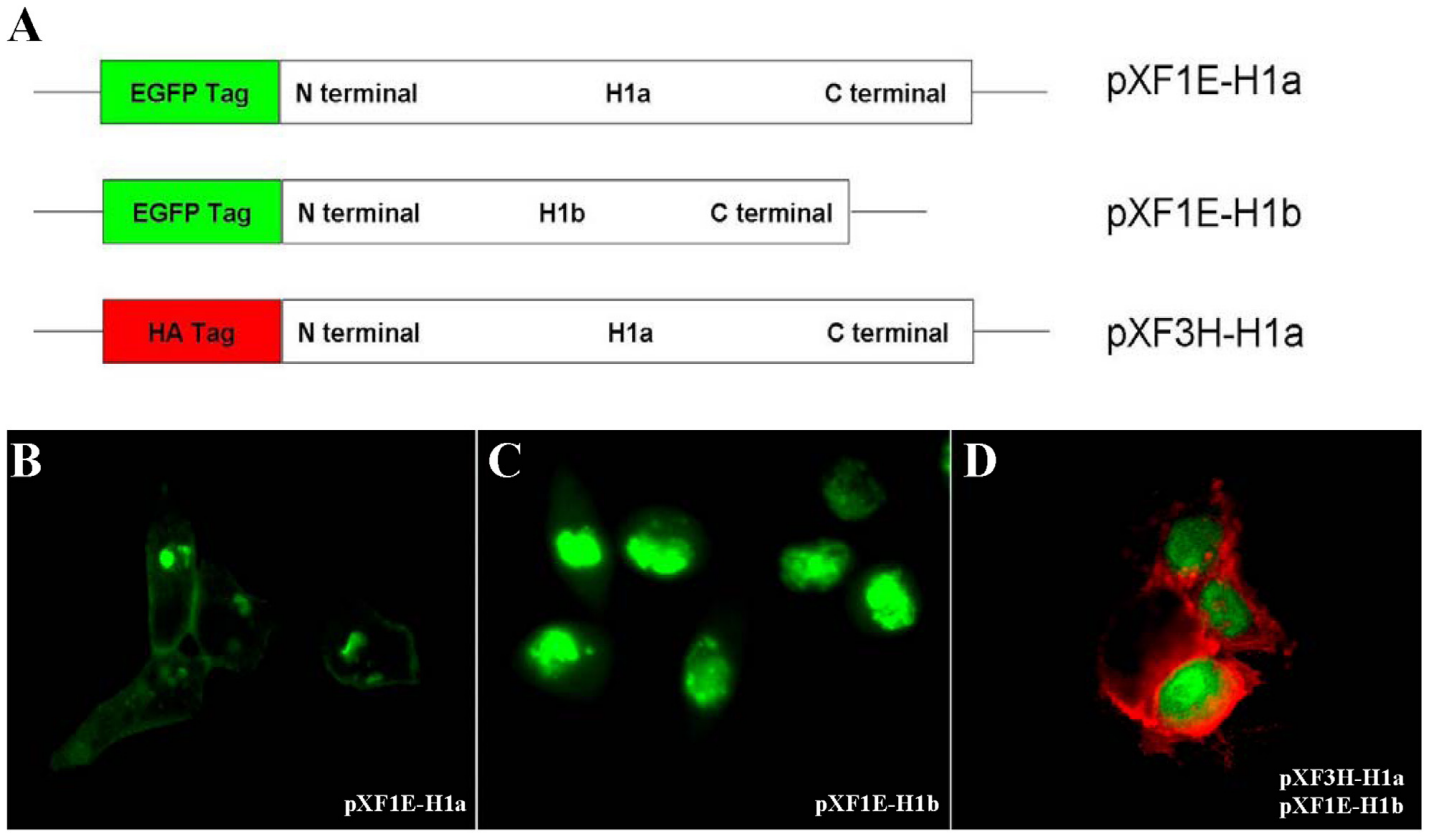
Localization of ASGPR H1b protein in transfected cells. (A) Fusion proteins of the two variants of ASGPR H1 subunit were designed to trace their cellular locations. Both ASGPR H1a and H1b were fused to EGFP in the vector pXF-1E. In pXF-3H-H1a, ASGPR H1a was expressed with an N-terminal HA tag. (B) Transient expression of EGFP-ASGPR H1a in HeLa cells by transfection with the plasmid pXF1E-H1a. Fluorescence signal of both the surface of the cellular membrane and the cytoplasm was observed. (C) Transient expression of EGFP-ASGPR H1b in HeLa cells by transfection with the plasmid pXF1E-H1b. The EGFP-H1b protein is only located in the cytoplasmic compartment. (D) Transient co-expression of EGFP-ASGPR H1a and H1b in HeLa cells by transfection with plasmids pXF3H-H1a and pXF1E-H1b. After co-transfection with both plasmids, HeLa cells were stained with anti-HA antibody and corresponding PE-labeled second antibody to detect ASGPR H1a.

We further addressed the question whether ASGPR H1b is synthesized in hepatocytes by IHC. Paraffin sections of human liver tissue were stained with anti-H1 and anti-H1b primary antibodies. The anti-H1 antibody, which recognizes both ASGPR H1a and H1b, stained proteins both at the surface of the cellular membrane and in the cytoplasm (Fig. 2A). By contrast, the anti-H1b antibody only stained proteins in the cytoplasm (Fig. 2B). This indicates that ASGPR H1b is produced in hepatocytes but does not accumulate at the cellular membrane, consistent with the fact that the transmembrane domain is missing in ASGPR H1b. These results were confirmed by the transient expression of H1a and H1b in Hela cells. The recombination protein H1a-EGFP was located in the plasma membrane (Fig. 3A), while H1b-EGFP can only be detected in the cytoplasm (Fig. 3B). Coexpression of both H1a-HA and H1b-EGFP further displayed that these two H1 variants had different localizations in cells (Fig. 3C).

### ASGPR H1b is present in human serum and in the supernatant from HepG2 cells

**Figure 4:**
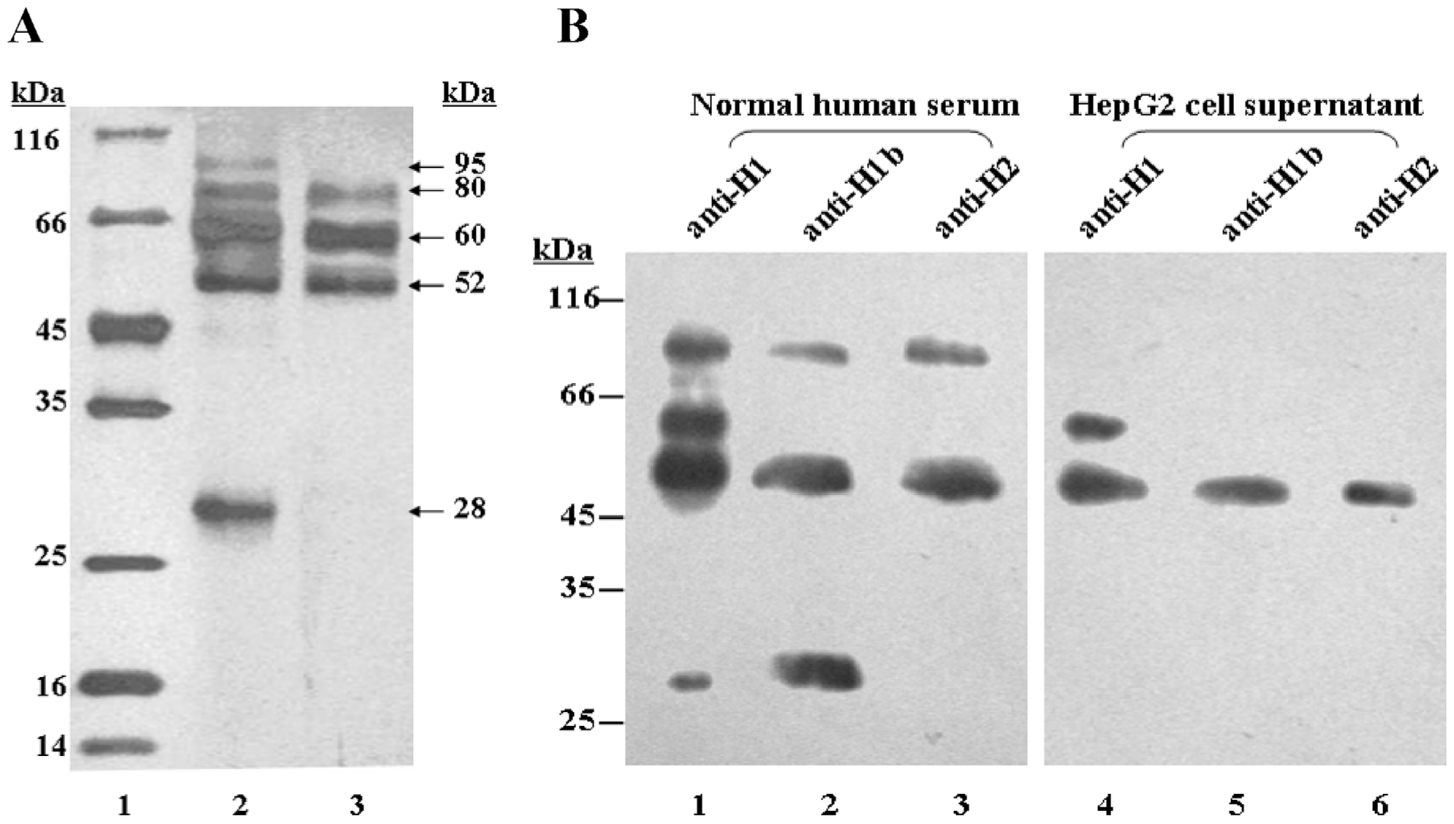
Analysis of functional sASGPR in serum and supernatant of HepG2 cells by SDS-PAGE and western blot. (A) SDS-PAGE. Lane 1: protein marker. Lane 2: functional sASGPR purified from normal human serum. Lane 3: functional sASGPR purified from supernatant of HepG2 cells. The positions of the fractions are indicated by arrows. (B) Western blot. Functional sASGPR purified from normal human serum (Lanes 1–3) and supernatant of HepG2 cells (Lanes 4–6) were separated by SDS-PAGE. Primary antibodies used are as follows: lanes 1 and 4: anti-H1; lanes 2 and 5: anti-H1b; lanes 3 and 6: anti-H2.

The lack of a transmembrane domain in ASGPR H1b suggests that this protein may be secreted from the cells. Therefore, we attempted to purify sASGPR from normal human serum and the supernatant of HepG2 cells by lactose-agarose affinity chromatography. The lactose-agarose affinity column-bound fractions were subjected to SDS-PAGE and Western blot analysis (Fig. 4). ASGPR H1b-positive proteins or protein complexes purified from human sera had molecular weights of approx. 28 kDa, 52 kDa, 60 kDa, 80 kDa, and 95 kDa in silver-stained gels (Fig. 4A). The fraction purified from HepG2 cell supernatants contained proteins and complexes with molecular weights of approx. 52 kDa, 60 kDa, and 80 kDa. The components of sASGPR were further analyzed by Western blot with antibodies specific for H1b (anti-H1b), H1a and H1b (anti-H1), and ASGPR H2 (anti-H2, Abnova, Taipei, Taiwan) (Fig. 4B). A protein with the molecular weight of 28 kDa was detected by anti-H1b and anti-H1. All three antibodies recognized the 52 kDa and 95 kDa proteins. A protein band at 60 kDa was recognized only by anti-H1 (Fig. 4B). It has been reported previously that the subunits of ASGPR form protein complexes that are stable even after denaturation in SDS-PAGE sample buffer containing SDS and β-mercaptoethanol. Therefore, we interpret the banding pattern on SDS-PAGE as follows: the protein band at 28 kDa represents the monomeric form of ASGPR H1b. The bands at 52 kDa and 95 kDa appear to be heterodimeric complexes of ASGPR H1b and H2. The remaining band, at 60 kDa, likely represents a homodimer of ASGPR H1a. However, the bands at 95 kDa and 28 kDa were not detected in HepG2 cell culture supernatants (Fig. 4B).

### Binding of ASOR to ASGPR-expressing cells is reduced in the presence of sASGPR

**Figure 5:**
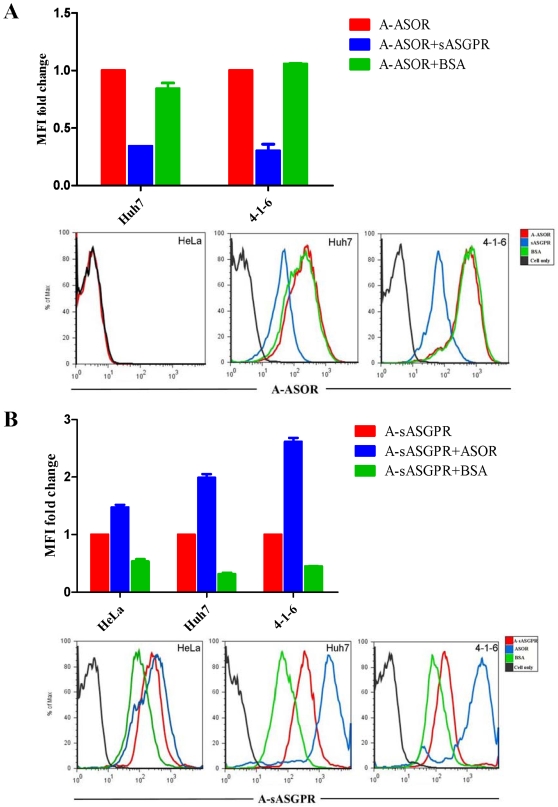
Ligands binding assay. The binding of fluorescence labeled A-ASOR (A) and A-sASGPR (B) to cells was determined by FACS. (A) Huh7 cells and cell line 4-1-6 were incubated with A-ASOR alone, or with A-ASOR in the presence of s-ASGPR or BSA. Binding of A-ASOR were determined by MFI. HeLa cells were used as a negtive control. The MFI value of the sample with A-ASOR alone was set as 100%. (B) HeLa cells, Huh7 cells, and the ASGPR-expressing cell line 4-1-6 were incubated with A-sASGPR alone, or with A-sASGPR in the presence of ASOR or BSA. Binding of A-sASGPR was determined by MFI. The MFI value of the sample with A-sASGPR alone was set as 100%.

To determine whether the presence of the soluble form of ASGPR affects the function of membrane-bound ASGPR, Huh7 cells and the stably transfected cell line 4-1-6 expressing membrane-bound ASGPR were incubated with Alexa Fluor 647-labeled ASOR (A-ASOR) alone, or with A-ASOR pre-incubated with s-ASGPR or BSA. Compared with adding A-ASOR alone, the binding of ASOR to cells was significantly reduced in the presence of sASGPR in the medium, while no change of ASOR binding was observed in the presence of BSA. HeLa cells were used as a negtive control and did not show any binding of A-ASOR (Fig. 5A). Over expression of H1b alone in Huh7 cells or expression of H1b alone in 4-1-6 cells by H1b expression plamid transfection showed no effect on ASOR binding to cells (Figure S3). These results suggest that sASGPR as a complex is able to bind to ASOR, and pose a competition to the binding between membrane ASGPR and ASOR.

### Binding of sASGPR to ASGPR-expressing cells was increased in the presence of ASOR

We next addressed the question of whether sASGPR itself can bind to membrane-bound ASGPR expressing cells or not. HeLa cells, Huh7 cells and cell line 4-1-6 were incubated with Alexa Fluor 647 labeled sASGPR (A-sASGPR) alone or with A-sASGPR combine ASOR or BSA. All cells showed a nonspecific binding with A-sASGPR since this binding could be significantly blocked by adding BSA to the system (Fig. 5B). Interestingly, the binding of sASGPR to Huh7 and 4-1-6 cells increased significantly by adding ASOR to the system (Fig. 5B). However, the binding of sASGPR to Hela cells did not change in the presence of ASOR. These results suggest that ASOR is able to enhance the binding of sASGPR to cells expressing membrane-bound ASGPR. It is likely that sASGPR and ASOR form complexes, which are able to interact with membrane-bound ASGPR on the cell surface.

### H1b expression is reduced in HepG2.2.15 cells and HCV-infected Huh7.5.1 cells

**Figure 6:**
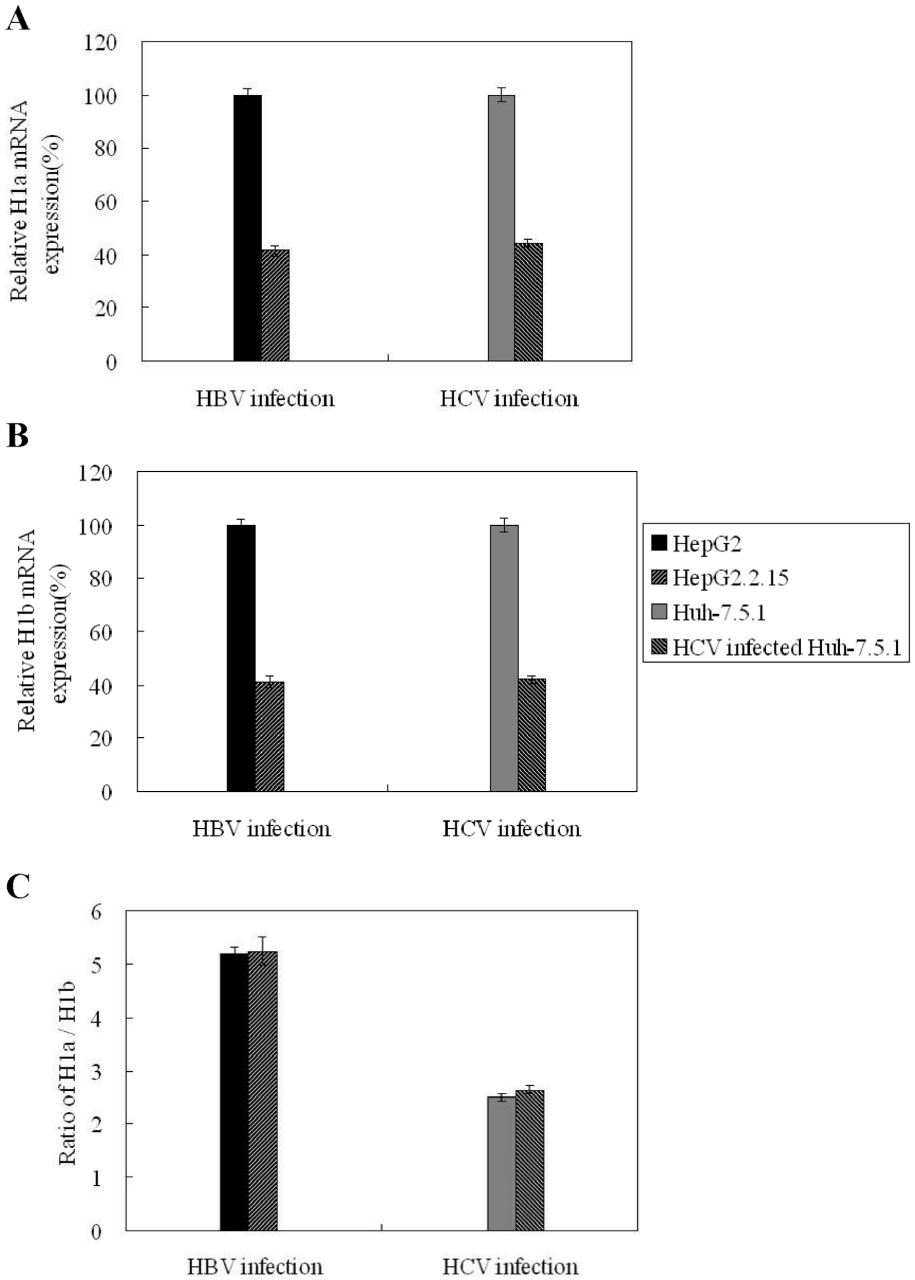
ASGPR H1b expression in hepatoma cells with and without viral replication. The expression levels of ASGPR H1a (A) and H1b (B) transcripts were determined by real-time PCR. The mRNA levels of ASGPR H1 variants in HepG2 cells HepG2.2.15 with HBV replication, Huh7.5.1 cells, and HCV infected Huh7.5.1 cells were determined. The expression levels of ASGPR H1 in HepG2 and Huh7.5.1 cells were set as 100%. (C) The ratio of H1a/H1b expressed in hepatoma cells.

Previous reports indicated that ASGPR levels are decreased in some liver diseases [17]. To determine whether there were any changes in H1a and H1b expression in different liver diseases, we investigated the expression levels of H1 variants in different hepatoma cells with real-time PCR (Fig. 6A and 6B). Both H1a and H1b expression were decreased by approx. 60% in HepG2.2.15 cells with active HBV replication compared to HepG2 cells. Similarly, a reduction of H1a and H1b expression was measured in HCV-infected Huh7.5.1 cells compared to uninfected Huh7.5.1 cells. HBV gene knockdown by RNAi partially restored H1a and H1b expression in HepG2.2.15 cells (data not shown). Further, HepG2 cells have a higher H1a/H1b ratio than Huh7 cells and liver tissues. Both HBV and HCV infection had no effect on the H1a/H1b ratio (Fig. 6C).

## Discussion

In this study, two distinct ASGPR H1 cDNAs, designated H1a and H1b, were cloned and sequenced. Sequence data suggest that these ASGPR H1 cDNAs represent splice variants of the ASGPR pre-mRNA. Compared with ASGPR H1a, a region of 117 nucleotides corresponding to the second exon of the ASGPR H1 genomic sequence is deleted in H1b, most likely by alternative splicing. At the protein level, the second exon encodes a transmembrane domain that is critical for membrane anchoring of the H1 protein [18], [19]. The lack of the transmembrane domain predicts that variant H1b is unable localize to the cell surface. Using a specific antibody that discriminates between ASGPR H1a and H1b, we detected ASGPR H1b protein in normal human sera and in the supernatant of hepatoma cells, indicating that it is secreted by these cells. Interestingly, the secreted forms of ASGPR H1 and H2 were found to co-exist in complexes and are apparently functional, because ASGPR proteins in sera and cell supernatants are able to bind to lactose-sepharose. In culture supernants of hepatoma cells HepG2, two bands representing sASGPR complexes at 28 kD and 95 kD were not detected. A possible reason of this observation is the very low expression level of H1b in HepG2 cells (Tab. 1). Only the major form consisting H1a, H1b, and H2 was produced, while other minor forms like the 28 kDa band (considered as the monomeric form of H1b) and the 95 kDa band (considered to be complexes of ASGPR H1b and H2) are apparently not synthesized at a sufficient level.

In previous work, ASGPR minor subunit H2 was shown to be secreted [9]. Both secreted forms of ASGPR H1 and H2 contain the stalk domains that are presumably required for hetero-oligomeric ASGPR complex formation [20]. It has been confirmed that the high affinity and specificity of binding to typical ASGP ligands are the result of multiple carbohydrate recognition domains in a hetero-oligomeric ASGPR complex binding to multiple complementary Gal/GalNAc residues in a glycan ligand containing multiple nonreducing termini [21], [22]. Though it was possible to synthesize and study homo-oligomeric ASGPR complexes, only ASGPR homo-oligomers could bind to typical ASGP ligands [23]–[27].

Secreted forms have been found for many membrane receptors [28]. In some cases, cleavage of the wild-type receptor occurs at the cell surface [29]. In other cases, alternative splicing creates truncated mRNAs encoding proteins that lack a transmembrane domain and are secreted [30]. In the case of the ASGPR H2a, alternative splicing creates a transcript containing an extra mini-exon, which plays a role in the cleavage and secretion of the ectodomain [9]. Alternative splicing is an effective mechanism for generating multiple protein isoforms with diverse functions from a single gene. In most cases, protein isoforms that arise from alternative splicing of a single gene transcript share extensive regions of identity and vary only in one or more specific domains, thus allowing the fine tuning of specific protein functions [31], [32].

**Figure 7:**
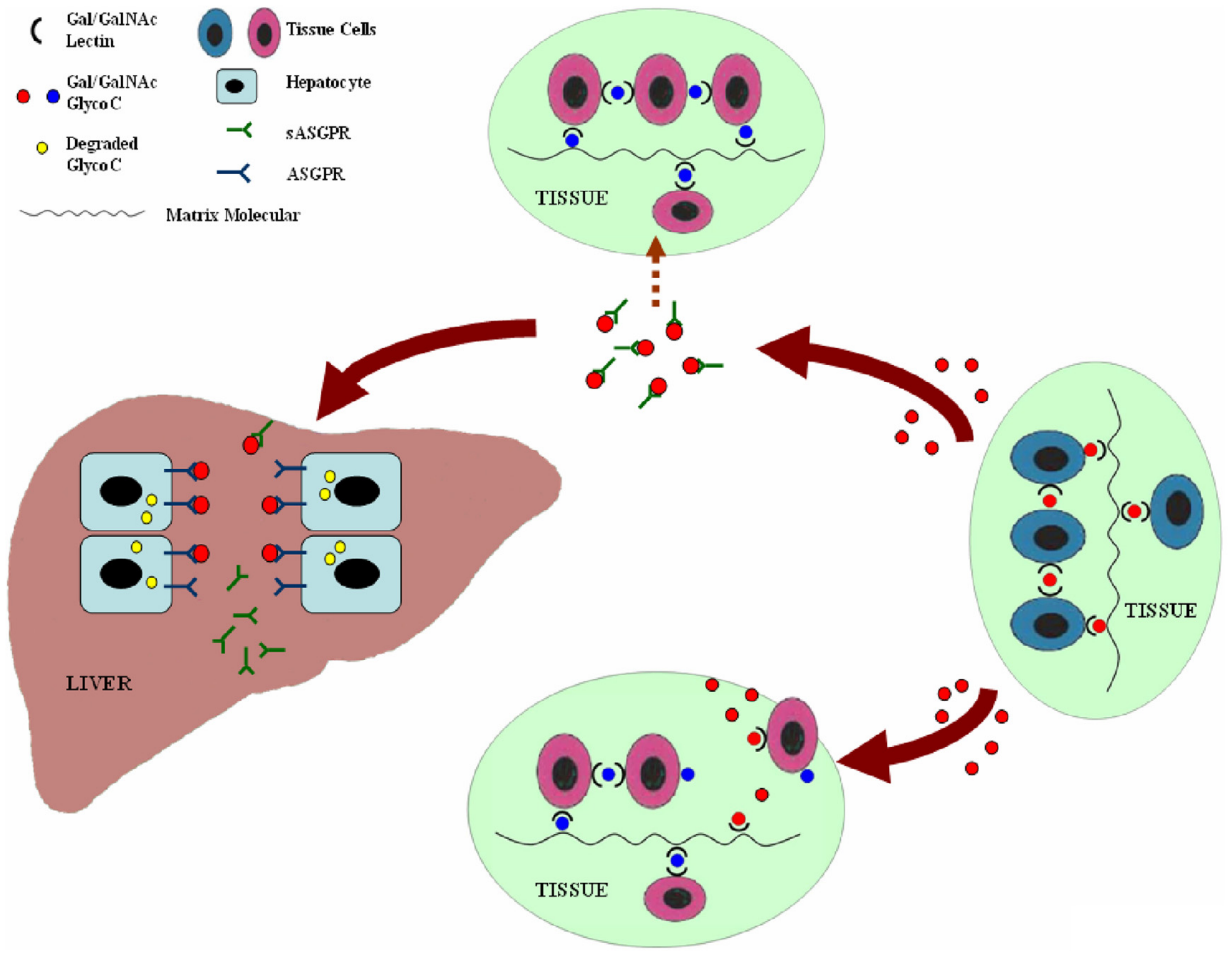
Possible physiological function of sASGPR. This diagram depicts the dynamic metabolic turnover of Gal/GalNAc glycoconjugates and their removal by the liver, as described by the Gal/GalNAc homeostasis hypothesis. Various tissues synthesize and secrete different Gal/GalNAc-specific lectins (black half circles) and Gal/GalNAc-containing glycoconjugates (red or blue full circles). The maintenance of these cell-cell or cell-matrix interactions requires the continuous participation of these molecules. Normal tissue turnover, injury, diet, disease, or other events may cause Gal/GalNAc glycoconjugates to be released into the blood. These soluble Gal/GalNAc glycoconjugates will be recognized and bound by sASGPR until they are transported safely to the liver, where they will be removed and degraded by the hepatic ASGPR system (molecules undergoing intracellular degradation are indicated by yellow full circles). Without the help of sASGPR, soluble Gal/GalNAc glycoconjugates will compete for the endogenous cell-cell or cell-matrix interactions between one or more Gal/GalNAc glycoconjugates and their lectin receptors before they can be removed by the hepatic ASGPR system. This competition could result in the disruption of normal tissue organization, cell growth regulation, or other functions necessary for normal healthy tissue homeostasis.

Despite extensive studies, the physiological function of ASGPR has not yet been established [33]. The most rational model for the function of ASGPR seems to be the galactosyl homeostasis hypothesis, proposed by Weigel [34]. This model recognizes that Gal- and GalNAc-containing glycoconjugates are important — and often critical — molecules in the wide array of cell-cell or cell-matrix interactions with many different cognate lectins in tissues throughout the body. Some of these lectin-glycoconjugate interactions likely occur during development, when ASGPR is not expressed. In adult tissues, the maintenance of these cell-cell or cell-matrix interactions requires a continuous participation of Gal/GalNAc-specific lectins or receptors with Gal/GalNAc glycoconjugates. Therefore, the introduction of any soluble Gal/GalNAc glycoconjugate (due to normal tissue turnover, injury, diet, disease, etc.) will compete for endogenous cell-cell or cell-matrix interactions between one or more Gal/GalNAc glycoconjugates and their lectin receptors. This competition can result in disruption of tissue organization, cell growth regulation or other homeostasis functions in tissues. The hepatic ASGPR system is proposed to be responsible for removing deleterious glycoconjugates from the circulation. In this light, sASGPR may be important to quickly bind soluble Gal/GalNAc glycoconjugates in the blood, prevent their interaction with normal cells and tissues until they are cleared in the liver (Fig. 7). This hypothesis of the function of sASGPR was supported by the results of our in vitro binding assay. The inhibition of ASOR binding to cells by sASGPR indicated that they can function in binding asialoglycoproteins in the plasma and preventing their interaction with the cell surface receptor, thus prolonging their life time in the circulation. The increased sASGPR binding to cells by ASOR further demonstrated that ASOR-sASGPR complex can still be captured by membrane ASGPR of hepatocytes and finally be removed from the circulation.

Hepatic ASGPR is thought to be a potential liver-specific receptor for many viruses [35]–[38]. Antibodies to ASGPR were found in more than 80% of patients with chronic-active autoimmune-hepatitis (AIH), more than 70% of patients with hepatitis B, and about 20% of patients with primary biliary cirrhosis. The titer of ASGPR antibody in patients with AIH correlates with biopsy-proven periportal inflammatory activity [39]–[41]. An sASGPR was found to facilitate hemolysis in patients with alcoholic cirrhosis of the liver [42]. Therefore, the function of sASGPR in human diseases should also be assessed. As shown in Fig. 6, the expression of ASGPR H1b transcripts in HBV- or HCV-infected cells was significantly reduced compared to uninfected cells. This suggests that the expression of sASGPR may inversely correlate with the pathology of hepatitis virus infection.

## Supporting Information

Figure S1 Sequence analysis and RT-PCR detection of splice variants of ASGPR H1. (A) Detection of ASGPR H1b transcript in HepG2 cells and Huh7 cells. Lane 1: DNA ladder. Lane 2: RT-PCR products of total RNA from HepG2 cells. Lane 3: RT-PCR products of total RNA from Huh7 cells. Lane 4: RT-PCR products of total RNA from HeLa cells. (B) Lane 1: DNA ladder. Lane 2: H1a detected in real time FQ-PCR. Lane 3: H1b detected in real time FQ-PCR.
(0.96 MB TIF)

Figure S2 Preparation and identification of polyclonal antibody recognizing ASGPR H1b specifically (anti-H1b). (A) Sequence comparison of ASGPR H1a and H1b. The 117 nt deletion in ASGPR H1b contains the entire transmembrane domain (TMD) and part of the cytoplasmic domain of ASGPR H1a. An H1b peptide SDHHQLRKDSQLQE corresponding to an ASGPR H1b specific sequence was synthesized for immunization to generate an anti-H1b antiserum. (B) Determination of the titer of anti-H1b antibody by indirect ELISA. The antiserum to the ASGPR H1b specific sequence was diluted serially and tested against the ASGPR H1b peptide and compared with a control serum. The anti-H1b serum was reactive against the peptide at a dilution over 1∶100,000. In contrast, the control serum was not reactive to ASGPR H1b peptide. (C) The specificity of anti-H1b antiserum. The anti-H1b serum was tested against ASGPR H1b peptide and an unrelated peptide. The anti-Hb1 shows no reactivity to the unrelated peptide, indicating the specificity binding to the H1b peptide of anti-H1b.
(0.80 MB TIF)

Figure S3 Over expression of H1b alone does not effect ASOR binding. Plasmid pcDNA3.1-H1b was transfected to Huh7 cells and cell line 4-1-6. Cells were then incubated with fluorescence labeled A-ASOR, The binding of A-ASOR to cells was determined by FACS. Binding of A-ASOR was determined by MFI. The MFI value of the sample with A-ASOR alone was set as 100%.
(0.01 MB TIF)
