# Supplementary figures and images for: A New Splice Variant of the Major Subunit of Human Asialoglycoprotein Receptor Encodes a Secreted Form in Hepatocytes
Source: PLoS One. 2010 Sep 23;5(9):e12934. doi: 10.1371/journal.pone.0012934 (PMC2944864; doi:10.1371/journal.pone.0012934)

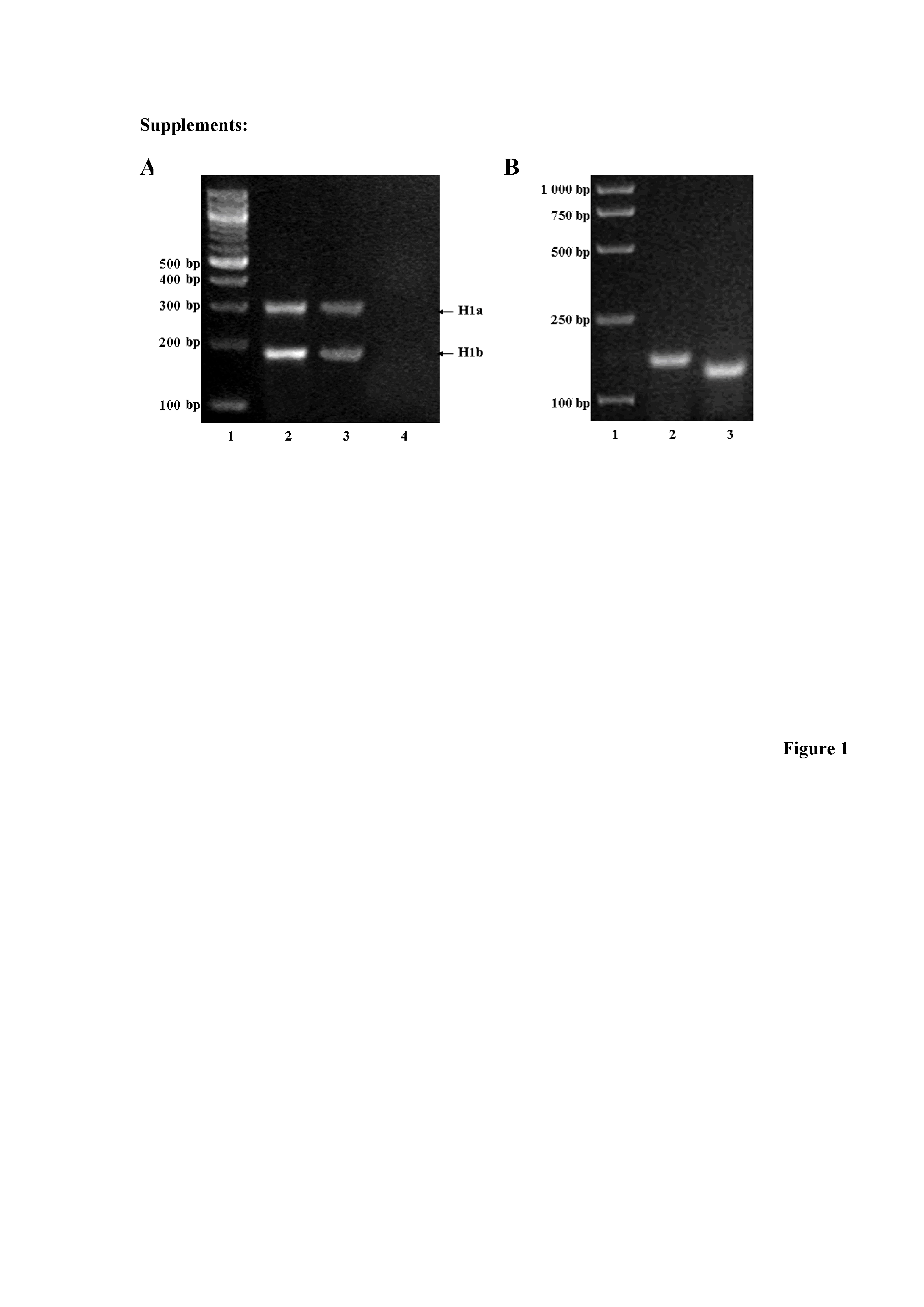

Supplement: Figure S1 — Sequence analysis and RT-PCR detection of splice variants of ASGPR H1. (A) Detection of ASGPR H1b transcript in HepG2 cells and Huh7 cells. Lane 1: DNA ladder. Lane 2: RT-PCR products of total RNA from HepG2 cells. Lane 3: RT-PCR products of total RNA from Huh7 cells. Lane 4: RT-PCR products of total RNA from HeLa cells. (B) Lane 1: DNA ladder. Lane 2: H1a detected in real time FQ-PCR. Lane 3: H1b detected in real time FQ-PCR. (0.96 MB TIF) [file pone.0012934.s001.tif]

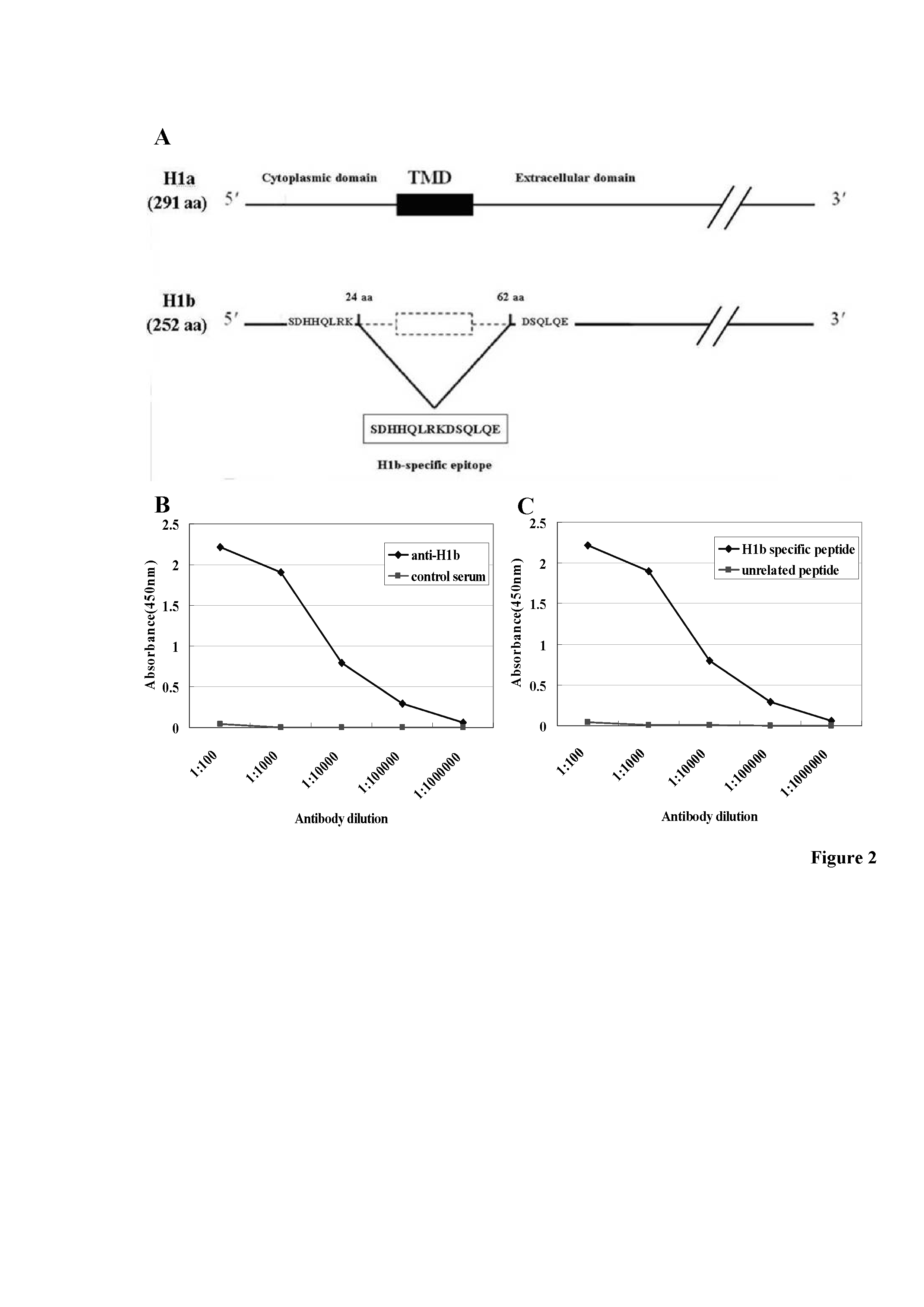

Supplement: Figure S2 — Preparation and identification of polyclonal antibody recognizing ASGPR H1b specifically (anti-H1b). (A) Sequence comparison of ASGPR H1a and H1b. The 117 nt deletion in ASGPR H1b contains the entire transmembrane domain (TMD) and part of the cytoplasmic domain of ASGPR H1a. An H1b peptide SDHHQLRKDSQLQE corresponding to an ASGPR H1b specific sequence was synthesized for immunization to generate an anti-H1b antiserum. (B) Determination of the titer of anti-H1b antibody by indirect ELISA. The antiserum to the ASGPR H1b specific sequence was diluted serially and tested against the ASGPR H1b peptide and compared with a control serum. The anti-H1b serum was reactive against the peptide at a dilution over 1∶100,000. In contrast, the control serum was not reactive to ASGPR H1b peptide. (C) The specificity of anti-H1b antiserum. The anti-H1b serum was tested against ASGPR H1b peptide and an unrelated peptide. The anti-Hb1 shows no reactivity to the unrelated peptide, indicating the specificity binding to the H1b peptide of anti-H1b. (0.80 MB TIF) [file pone.0012934.s002.tif]
